# Early Lung Cancer Diagnosis by Biosensors

**DOI:** 10.3390/ijms140815479

**Published:** 2013-07-25

**Authors:** Yuqian Zhang, Dongliang Yang, Lixing Weng, Lianhui Wang

**Affiliations:** 1 Key Laboratory for Organic Electronics & Information Displays (KLOEID) and Institute of Advanced Materials (IAM), Nanjing University of Posts and Telecommunications, 9 Wenyuan Road, Nanjing 210023, China; E-Mails: kekedemajiao@live.cn (Y.Z.); luck1023@163.com (D.Y.); 2 College of Geography and Biological Information, Nanjing University of Posts and Telecommunications, 9 Wenyuan Road, Nanjing 210023, China

**Keywords:** lung cancer, early diagnosis, biomarker, microRNA, tumor-associated antigen, biosensor

## Abstract

Lung cancer causes an extreme threat to human health, and the mortality rate due to lung cancer has not decreased during the last decade. Prognosis or early diagnosis could help reduce the mortality rate. If microRNA and tumor-associated antigens (TAAs), as well as the corresponding autoantibodies, can be detected prior to clinical diagnosis, such high sensitivity of biosensors makes the early diagnosis and prognosis of cancer realizable. This review provides an overview of tumor-associated biomarker identifying methods and the biosensor technology available today. Laboratorial researches utilizing biosensors for early lung cancer diagnosis will be highlighted.

## 1. Introduction

Lung cancer is the most common cause of cancer-related death worldwide. In 2008, approximately 1.6 million new lung cancer cases and 1.4 million lung cancer deaths have occurred all over the world [1,2]. The mortality rate has not decreased during the last decade [1,3,4], because the lack of clinical symptoms in early-stage lung cancer leads to diagnosis at a late stage [5]. A population-based experiment showed that lobectomy was the best long-term outcome in fit elderly patients but only with the early stage non-small cell lung cancer (NSCLC) [6]. NSCLC has an overall 5-year survival of less than 15%, while the 5-year survival for stage I disease is over 50%. However, 75% of NSCLC is diagnosed at an advanced stage which is not amenable to surgery [7]. Thus, there is a great need to detect cancer at an early stage, ideally before invasion. This has led to significant interest in effective screening methods to detect early-stage cancers, particularly for high-risk groups, such as current or former smokers [8].

However, the aggressive and heterogeneous nature of lung cancer has thwarted efforts to reduce mortality through the use of screening [9]. Annual screening with chest radiograph and sputum cytologic analysis failed to reduce mortality, and so did the meta-analysis [10]. Nowadays, low-dose computed tomography (LDCT) is widely used for lung cancer screening [11], and reduces lung cancer mortality by 20% and overall mortality by 7% in the group randomly assigned to an annual spiral CT scanning compared to chest radiographsm [9]. A large number of studies focus on the amelioration of CT technology. 18F-Fluorodeoxyglucose positron emission to mography/computed tomography (18F-FDG PET/CT) is routinely used in oncological imaging [12–15]. However, these currently available techniques still do not fulfill the requirements for reliable discrimination between early lung cancer and healthy subjects. What’s worse, the false positive result ratio of the LDCT screening is as high as 96.4% [9], and there is also variability in the reported accuracy of 18F-FDG PET/CT [13], which may result in an increased morbidity from unnecessary surgical treatment and also serious psychological burden to the patients and the families.

Tumor growth is accompanied by gene and protein changes. Such methylation or point mutation of DNA, RNA and mutated or aberrantly expressed proteins, carbohydrates, cytokines and chemokines, as well as volatile organic compounds from the peroxidation of the cell membrane species [16–18], could be detected months or years prior to clinical diagnosis and are able to act as cancer biomarkers. Zhong *et al.* have proved that tumor-associated autoantibodies for NSCLC could be detected 5 years before it could be detected using autoradiography [19]. These biomarkers have attracted considerable attention. In this paper, we mainly review these lung tumor-associated microRNAs (miRNAs) and antigens (TAAs), as well as their corresponding autoantibodies and screening methods.

The most frequent method to test serum TAAs is ELISA and solution hybridization detection method for miRNA currently, which are time consuming and expensive, and are not sufficiently sensitive for the low marker concentrations at early cancer stages [20]. Over the past decades, many groups have studied advanced technology to increase the sensitivity and specificity of biomarker-testing. A biosensor is a bioanalytical device incorporating a molecular recognition entity associated with a physicochemical transducer, providing advanced platforms for biomarker analysis [21]. To detect TAAs, monoclonal antibodies and aptamers are usually used as capture agents, and for miRNA, capture agents are usually corresponding ssDNA. A transducer is a device that converts recognition signal events into electrical signals. The transducer could be electrochemical (amperometry, potentiometry, conductimetry/impedimetry), optical (colorimetric, fluorescence, luminescence, interferometry), calorimetric (thermistor), or mass change (piezoelectric/acoustic wave), and are required to offer high throughput, high signal-to-noise ratio, relatively low instrumentation costs, good resolution and reproducible results. Besides the capture agent and transducer, surface chemistry for immobilization of capture agents onto solid surfaces should also be considered. Here, an overview of the potential biomarkers and biosensors for early diagnosis of lung cancer is presented, with an emphasis on the serum biomarker detection.

## 2. Lung Cancer Biomarkers

These mutated or aberrantly expressed microRNAs and proteins, as well as other compounds [21–23] and cancer cells [16,22], could be detected months or years prior to clinical diagnosis and be able to act as cancer biomarkers. Tumor biomarkers were initially used to support the diagnosis of cancer, yet now they are trend to predict the disease and also measure the pharmacodynamic effect of drugs. In view of non-invasive diagnosis of cancer at early stage sensitively, serum miRNA and tumor-associated antigen or autoantibody prior to the onset of cancer are the most frequently studied.

### 2.1. MicroRNA

MicroRNAs, the small non-coding RNAs with a length of 17–25 nt [23–25], regulate diverse biological processes at a post-transcriptional level. Recent evidence has shown that mutational, mis-expressed or altered miRNAs are implicated in the initiation and progression of human cancer [24,26]. miRNAs have been demonstrated to be present in the serum and plasma. Even more attractively, miRNA biogenesis maintains them in a protected state [26], so the levels of miRNAs in serum are stable, reproducible, and consistent among individuals of the same species, allowing the detection of miRNA directly from serum. Also, specific expression patterns of serum miRNAs for cancer have been identified, providing evidences that serum miRNAs contain fingerprints for cancers [25]. miRNAs prove useful in the prognosis, diagnosis, and staging of cancers as non-invasive biomarkers. Profiling has been exploited to identify miRNA as disease fingerprints. In addition, altered precursor miRNA could affect miRNA processing, expression, and target mRNA binding [27].

Appling microassay techniques, mostly the Solexa sequencing technique, the miRNA expression is analyzed. MiRNAs that are statistically different from health samples will be identified. The solution hybridization detection method and/or real-time quantitative RT-PCR analysis are employed to validate the results. Finally, the expression patterns of serum miRNAs for different diseases are obtained [23,25,27,28]. The mechanisms of miRNA release into the bloodstream, the putative functional significance and the miRNA identification in lung cancer have been reviewed [29]. Another review overviewed *miR-29*, a miRNA family consisting of three mature members, *miR-29a*, *miR-29b* and *miR-29c*. As a cancer suppression, *miR-29* is silenced or down-regulated in many different types of cancer. Furthermore, the *miR-29* family can be activated by interferon signaling, especially in response to viral infections [30]. Lung carcinogen NNK exposure was demonstrated to be capable of changing the expression of serum miRNAs. Serum *miR-206* and *miR-133b* could be associated with lung carcinogenesis induced by NNK and might be potential biomarkers for lung carcinogenesis [31]. High *hsa-mir-155* and low *hsa-let-7a-2* expression were identified correlated with survival of lung cancer [28]. *MiR-449c* downregulation in NSCLC was investigated, and the overexpression of *miR-449c* could suppress tumor growth *in vivo*. In addition, *c-Myc* was identified as a direct target gene of *miR-449c* [32]. Circulating *miR-142-3p* and *miR-29b* were identified using qRT-PCR and confirmed to be increased in sera of early-stage adenocarcinoma patients [33]. The results of some studies are inconsistent due to different technological platforms and small sample sizes. For further confirmation, a comprehensive meta-analysis of 20 published miRNAs in lung cancer was performed. Seven upregulated (*miR-21*, *miR-210*, *miR-182*, *miR-31*, *miR-200b*, *miR-205* and *miR-183*) and eight downregulated (*miR-126-3p*, *miR-30a*, *miR-30d*, *miR-486-5p*, *miR-451a*, *miR-126-5p*, *miR-143* and *miR-145*) miRNAs were identified [34].

We conclude that serum miRNAs can serve as potential biomarkers for the detection of various cancers and other diseases. A great number of groups are working on the identification of tumor-associated miRNAs, many of which are not discussed here. Both silence and overexpression of miRNAs may be involved in the development of cancer, while the overexpressed miRNAs are more suitable for early diagnosis or prognosis, which have the potential to revolutionize present clinical management.

### 2.2. Tumor-Associated Antigens (TAAs)

Cancer onset and progression are accompanied by mutated or aberrantly expressed proteins which would evoke immune response, resulting in the production of autoantibodies. These antibodies in cancer patients, but not in healthy human, could be detected months or years before the clinical diagnosis of cancer [35,36]. Tumor-associated antigens (TAAs), and their corresponding autoantibodies could serve as biomarkers for early cancer diagnosis or prognosis [16,35–39]. Luna Coronell J.A. *et al.* and Casiano C.A. *et al.* have reviewed the current status of tumor-associated antigens, and tumor-associated autoantibodies [39,40]. Two criteria used to establish the usefulness of biomarkers are sensitivity and specificity [41].

Serum TAAs and anti-TAA autoantibodies testing is supposed to be simple and fast, theoretically using only a few microliters of serum [39]. However, the use of TAAs for early diagnosis of cancer with the available clinical information is still insufficient because of their low initial concentration and heterogeneity in blood of cancer patients [40]. To solve the problem, TAA arrays comprising several antigens have been proposed, which significantly increase this frequency and hold great promise for the early detection of cancer [40]. Another developing technique focuses on the detection of immune response to tumor antigens. Serum antibodies are more stable than the antigens, and may be more abundant and readily detectable, especially at early cancer stages [42].

#### 2.2.1. Identification of TAAs

To discover new TAAs, the following methods are most frequently employed: SERPA (Serological Proteomic Analysis, also known as Proteomes or SPEAR, Serological and Proteomic Evaluation of Antibody Response), phage display libraries, SEREX (Serological Analysis of Recombinant cDNA Expression Libraries), and protein array-based technology [39,43,44]. There are three main points of all these methods screening for TAAs: getting the proteins directly from the tumor tissues or serum, or expressed by a cDNA library, subsequent different separating techniques like two-dimensional (2D) gel electrophoresis or 2D liquid chromatography, and screening tumor-associated proteins using serum antibodies from cancer patients.

##### 2.2.1.1. SERPA

These methods identifying TAAs all start from extracting protein or mRNA from fresh tumor samples or cancer patients’ serum. Protein extracts can be used for SERPA. SERPA takes advantage of the classical two-dimensional gel electrophoresis (2-DE), western blotting and mass spectrometry (MS). Protein extracts are separated by 2-DE, transferred onto membranes by electroblotting and subsequently probed with sera from healthy individuals or cancer patients, which are compared and then the tumor-associated antigenic spots are identified by MS, which identifies the nature and abundance of the total proteins extracted [43].

Through SERPA, a protein gene product 9.5 (PGP 9.5) was detected in sera from patients with lung adenocarcinoma in two separate studies [45,46]. The sensitivity of PGP 9.5 and annexin I and II in patients with lung adenocarcinoma was 14%, 30% and 33% respectively. Another study using SERPA also detected PGP 9.5 from lung cancer cell line with a sensitivity of 55% and a specificity of 95% [47]. Li C. *et al.* identified nine proteins up-regulated in human lung squamous carcinoma (hLSC) [48]. They also performed the SERPA of human lung squamous carcinoma cell line HTB-182 and characterized 16 differentially expressed proteins which reacted with lung squamous carcinoma patients’ sera while not reacting with the control [49].

##### 2.2.1.2. Phage Display

Phage display is based on the construction of cDNA library. cDNAs obtained through reverse transcription with mRNA purified from fresh tumor samples or cell lines are cloned into prokaryotic-or eukaryotic expression systems, and organized into a phage-display library. Therefore, antibody fragments are expressed as fusions to the phage coat proteins and can be displayed on the bacteriophage surface, which will be used for identifying and isolating, and such phages carrying peptides with high affinity and specificity to given target molecules will be purified [39,50].

The selection of antibodies from phage libraries consists of two steps: panning and screening. Through the expression of cDNAs, peptides from the tumor or cell line are expressed as fusions with phage proteins and displayed on the phage surface. During panning, library phage preparations are incubated with the antigen of choice, unbound phage are discarded and remaining phage recovered. Recovered phage are subsequently amplified by infecting *E. coli* and further rounds of panning are applied, yielding a polyclonal mixture of phage antibodies enriched for antigen-specific binders. In the screening process, the polyclonal mixtures are converted into monoclonal antibodies. Finally, *E. coli* cells are infected with the phages, and then plated on selective plates, and single colonies are picked. Thus, highly specific, monoclonal antibody clones are obtained, from which the antibody genes can be readily isolated for further analysis.

Zhong L. *et al.* screened T7-phage NSCLC cDNA libraries, and five most predictive antibody markers achieved 91.3% sensitivity and 91.3% specificity [19]. A six-phage peptide detector was validated to be able to discriminate between NSCLC patients and healthy controls with a sensitivity and specificity of >92%, and had similar validity for indicating NSCLC at early stage. The seroreactivity of the six phage peptides in the patients with chemotherapy and the COPD was not perceptible, indicating that the six-phage peptide detector may not stand out for NSCLC relapse after chemotherapy [51].

##### 2.2.1.3. SEREX

SEREX was first developed in 1995 [52]. The first step of SEREX is also the construction of cDNA libraries. Subsequent steps include plating phages, transferring onto a nitrocellulose membrane, induction of protein expression, screening, and finally identifying and sequencing individual reactive clones [53]. Almost 3000 of these autoantigens are documented in a public access online database known as the Cancer Immunome Datebase (CID) http://ludwig-sun5.unil.ch/CancerImmunomeDB/ [54]. Using the lambda-ZAP vector, cDNA expression library was constructed and three antigens (L-8, L-19, L-51) showed higher positive rates in lung cancer patients than in health donors [55]. Another SEREX found 4 *SOX* group B genes (*SOX1*, *SOX2*, *SOX3*, and *SOX21*) and *ZIC2* expressed in sera of SCLC patients. SOX1 and/or SOX2 were the main antigens eliciting anti-SOX responses [56].

##### 2.2.1.4. Protein Microarrays

Protein array technology could screen the humoral immune response in cancer against thousands of recombinant-, fractions- or purified proteins, as well as synthetic peptides and even unknown proteins in a high throughput [43,57]. Protein microarrays of tumor-derived proteins could profile the antibody repertoire in the sera of cancer patients and controls. Proteins are separated through 2D liquid chromatography and then spotted in microarray on coated microscope slides, which are then incubated individually with serum samples from cancer patients and controls. The amount of immunoglobulin bound to each fraction could be quantified, and reflect an immune response. Protein microarray technology should not only aid in improved diagnostics, but has already contributed to the identification of complex autoantibody signatures that may represent disease subgroups [57,58]. The reproducibility of natural protein microarrays and their ability to distinguish lung cancer sera have been assessed and the result was affirmative [59]. Madoz-Gurpide J. *et al.* combined liquid phase protein separations with microarray technology [60].

##### 2.2.1.5. Reverse Capture Microarray

The “reverse capture” autoantibody microarray was developed to study such antigen-antibody reactivity as recombinant proteins or synthetic peptides, because they might fail to accurately detect autoantibody binding due to the lack of post-translational modifications (PTMs) [61,62]. The reverse capture microarray is based on the dual-antibody sandwich immunoassay platform of enzyme-linked immunosorbent assay (ELISA), which allows the antigens to be immobilized in their native configuration [61]. Then the array is incubated with labeled IgG from cancer or control samples, and performs a two-slide dye-swap to account for any dye effects [62]. The reverse capture microassay protocol could facilitate the detection of autoimmunity to native host antigens and also has the advantage over traditional protein arrays of being able to detect autoimmunity to epitopes found on the PTMs of native antigens. The process can be completed in 9–10 h [63]. These developments enable the immediate use of high-density antibody and protein microarrays in biomarker discovery studies. The substrate for reverse capture microarray was studied, and the hydrogel substrate yielded a higher signal-to-noise ratio than the others [64]. However, only known antigens which are commercially available can be analyzed. Post-translationally modified antigens cannot be differentiated.

##### 2.2.1.6. Multiple Affinity Protein Profiling (MAPPing)

MAPPing is such an approach combining 2-D immunoaffinity chromatography, enzymatic digestion of the isolated antigens, and nano flow separation of the resulting peptides. By these approaches, both proteins recognized by autoantibodies independently of a cancer status, and a limited number of proteins reacting preferentially with cancer sera will be identified [65]. Nonspecific TAAs in a cancer cell line or tumor tissue lysate bind to IgG obtained from healthy controls in the immunoaffinity column and are removed from the lysate, and then the other fraction which flows through the column will be subjected to the 2D immunoaffinity column that contains IgG from the cancer patients [66], and those that bind to the column are likely to be cancer-specific and will be eluted and identificated by tandem MS.

#### 2.2.2. TAAs of Lung Cancer

Protein gene product 9.5 (PGP 9.5) was detected in sera from patients with lung adenocarcinoma [45,46]. The sensitivity of PGP 9.5 and annexin I and II in patients with lung adenocarcinoma was 14%, 30% and 33% respectively. None of the healthy controls showed immunoreactivity against annexin I and II. However, autoantibodies against annexin II were found in patients with other cancers [45]. Immune response manifested by annexins I and II autoantibodies may occur commonly in lung cancer and is associated with high circulating levels of inflammatory cytokine [47]. At the same time, a 14-3-3 theta was found to exhibit significant reactivity with sera of lung adenocarcinoma patients. A panel of three proteins consisting of 14-3-3 theta, annexin I and PGP 9.5 proteins, gave a sensitivity of 55% at 95% specificity in discriminating lung cancer at the preclinical stage from controls [47]. Further study of the panel of annexin I, PGP9.5, and 14-3-3 theta antigens found that reactivity against PGP 9.5 was not significant, while annexin I, 14-3-3 theta, and a novel lung cancer antigen, LAMR1 showed significant reactivity for prediagnostic sera [67].

Eight antibodies, that reacted with lung cancer patients’ sera, but not with sera derived from lung tuberculosis or healthy controls, were isolated from A549 lung adenocarcinoma cell lysate [68]. Autoantibodies against alpha-enolase were detected in a subset of NSCLC patients’ sera, with the prevalence of 27.7% in patients with NSCLC [69]. Triosephosphate isomerase and superoxide dismutase autoantibodies were detected in sera from over 20% patients with LSC but none from the normal controls [70]. Leave-one-out validation of five antibody markers achieved 91.3% sensitivity and 91.3% specificity for NSCLC, and it is worthy of mentioning that they could be identified 5 years before it could be detected using autoradiography [19].

The p53 alteration is the most common alteration found in human cancer, and mutant p53 accumulates in the nucleus of tumor cells. Patients with various types of neoplasias have p53 antibodies in their sera. These antibodies are mostly IgG, corresponding to a secondary immune response [35], with a specificity of 96% [71]. However, the sensitivity of such detection is only 30%. Although the clinical value of these antibodies remains subject to debate, the finding of p53 antibodies in the sera of individuals with high risk of cancer (such as exposed workers or heavy smokers) [71], and the decrease of antibodies against p53 correlating with a good response to early therapy in lung cancer patients [35], indicate that they have promising potential in the early detection of cancer, as well as monitoring the therapies.

The detection of autoantibodies reactive to TAAs may not only identify cancer at an early stage, but also distinguish patients with different types of cancers. Cytokeratin18 (CK18) and villin1 were identified with autoantibodies in sera from two kinds of pulmonary carcinoma patients, adenocarcinoma (AD) and small cell carcinoma (SCC). CK18 could be observed in almost all lung cancer cases, while villin1 was detected in 38.6% of AD and 61.8% of large cell neuroendocrine carcinoma (LCNEC), respectively. Thus, villin1 and CK18 may be useful markers to distinguish LCNEC/AD from SCLC/SCC [72]. Another research showed that the NY-ESO-I autoantibody might also be used to distinguish patients with NSCLC from SCLC [73].

There are still limitations of TAAs. Because of the heterogenic nature of cancer, the autoantibody against a particular TAA could be found in only 10%–30% of patients [35,45–47,74]. Some TAAs may be nonspecific. Although a single autoantigen would lack adequate sensitivity and specificity, a panel of TAAs may overcome this problem by being detected simultaneously [43]. The presence of autoantibodies to a panel of seven TAAs (p53, c-myc, HER2, NY-ESO-1, CAGE, MUC1 and GBU4-5) was investigated with plasma from normal controls and patients with lung cancer. Normal individuals could be identified among NSCLC or SCLC with sensitivities ranging from 76% to 92%, and a specificity of 92% [75]. Some other TAA panels used for diagnosis of lung cancer are summarized in the Table 1.

## 3. Biosensors

**Figure 1:**
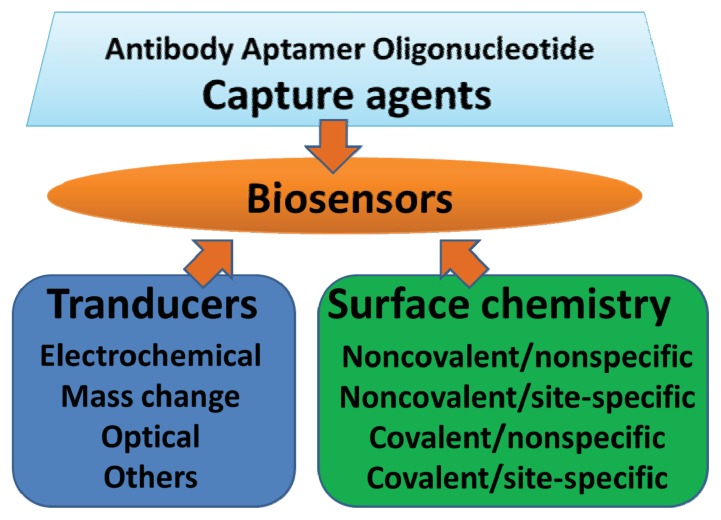
The three ingredients of biosensors.

A biosensor is a bioanalytical device incorporating a molecular recognition entity associated with or integrated with a physicochemical transducer [20]. Three ingredients should be considered when constructing a biosensor, as shown in Figure 1. And Table 2 shows some limit of detection (LOD) and linear range of the lung cancer biomarker detection using biosensors.

### 3.1. Capture Agents

The selection and production of the capture agents are critical in biosensor technology. ssDNA with high specificity and stability is applied as capture agents of miRNA. As for TAAs, antibodies and their fragments, antigens, fused proteins, aptamers, and molecularly imprinted polymers (MIPs), have been vigorously studied. With high affinity and specificity to target molecules, monoclonal or polyclonal antibodies are the most frequently used, followed by aptamers.

Antibodies were discovered at the end of the 19th century. They have been considered useful analytical tools since the monoclonal antibodies could be produced. Two methods are currently in use for the production of monoclonal antibodies. One is the immortalization of the antibody producer cells, which is achieved by Epstein-Barr virus transformation of a lymphocyte and/or by hybridoma generation. The other adapts molecular biology techniques as powerful and promising tools for the preparation, modification and improvement of monoclonal antibodies from either murine or human origin. Selected clones producing a monoclonal antibody can be cultured continuously theoretically. Thus, Monoclonal antibodies would be produced in sufficient quantities. Yet such antibodies are very expensive, as well as difficult to preserve. Also, the antibody-producing cells may die unexpectedly. The Antibody generation is difficult with molecules that are not well tolerated by animals for the process within an animal. The performance of the same antibody may vary from batch to batch. Various approaches are developed to circumvent these limitations, including displaying peptide libraries on phages or ribosomes, *in vitro* immunization, and taking aptamers substituting antibodies.

The development of the systematic evolution of ligands by exponential enrichment (SELEX) process isolates the oligonucleotide sequences and gives us the capacity to recognize virtually any class of target molecules with high affinity and specificity possible. These synthetic oligonucleotide (DNA or RNA) molecules with the length of 10 to 100 building blocks are named aptamers, derived from the latin *aptus*. Aptamers can be easily synthesized and amplified. The technique could involve screening very large combinatorial libraries of oligonucleotides by an iterative process of *in vitro* selection and amplification. A random oligonucleotide sequence library is obtained from combinatorial chemical synthesis. The library is incubated with a target of interest, and the target would tend to interact with several individual sequences, which would be isolated and amplified to obtain an enriched library. The selection/amplification process would take place for several times, and the ultima library is cloned and sequenced. Individual sequences are further characterized by their ability to bind to the target, and then truncated to the minimal target-binding domain, which is usually less than 40 nucleotides. A typical SELEX process may take approximately 2–3 months. Also, certain modifications at the 2′ position of the ribose ring would be made to keep RNA from nuclease. Cho H.S. *et al.* has reported a detection method using an aptamer sensor, targeting vascular endothelial growth factor-165 (VEGF165), a predominant biomarker for cancer angiogenesis [81]. The aptasensor provides felicitous sensitivity and specificity, with a linear rang from 25 pg/mL to 25 μg/mL, and good agreement within the limit of the ELISA kit in serum and saliva. McCauley T.G. *et al.* also developed an aptamer based biosensor for detection and quantification of multiplex cancer-associated protein analytes from human serum and cellular extracts [94], and the solid phase aptamer-protein interactions recapitulated binding interactions in solution. An aptamer has also been used for recognition and detection of cancer cells [95].

### 3.2. Surface Chemistry for Immobilization of Capture Agents onto Solid Surfaces

These proteins or oligonucleotides should be immobilized onto a slide coated with various surfaces in a planar or 3D platform, giving a stable layer of biomolecules without bio-activity being reduced significantly. Planar surface coating chemistries are categorized into four main groups based on the binding principle: nonspecific-noncovalent, nonspecifi-covalent, site specific-noncovalent and site specific-covalent [21]. Nonspecific immobilization approaches do not require any modifications of capture agents, and thereby facilitate capture-agent production. The substrates for noncovalent immobilization includes soft membranes such as poly(vinylidene difluoride) (PVDF) and glass slides modified with nitrocellulose [96] or poly(*L*-lysine), while the covalent protocol employs solid surfaces modified with aldehyde, epoxide or succinimidyl ester/isothiocyanate functionalities. The site specific-noncovalent immobilization techniques use certain affinity interaction molecules like streptavidin-biotin and His-tag-nickel-chelates. Moreover, self-assembly monolayers (SAMs) on gold coated surfaces are widely employed for site specific-covalent surface modification. All these coating chemistries should offer low background. Three dimensional (3D) platforms based on agarose hydrogel, polyacrylamide hydrogel, and sol-gel-encapsulated biomolecules patterned within multiwell PDMS films, as well as nanoparticles and beads have been developed, aiming to overcome the difficulty of limited spot density, increase signal to noise ratio, and avoid non-specific binging and cross-reactivity.

### 3.3. Transducers of Biosensors

A transducer is a device that converts recognition signal events into electrical signals, which could be electrochemical (amperometry, potentiometry, conductimetry/impedimetry), optical (colorimetric, fluorescence, luminescence, interferometry), and mass change (piezoelectric/acoustic wave), and so on. The transducer is required to offer high throughput, high signal-to-noise ratio, relatively low instrumentation costs, good resolution and reproducible results. The sensitivity and discrimination ability of a differential sensor are critically dependent on the number of sensing elements.

#### 3.3.1. Electrochemical Transducer

Liu J.H. and Ju H.X. [97], and Wang J. [98] have reviewed the electrochemical immunosensors, which have been miniaturized to small pocket size devices. For example, the glucose biosensor is applicable for home use, realizing rapid point-of-care measurement. Amperometric and potentiometric transducers are the most commonly used among all the electrochemical transducers. Amperometric biosensors could monitor the current associated with the reduction or oxidation of electroactivity. The potentiometric devices convert the biorecognition process into a potential signal in connection to the use of ion-selective electrodes (ISE). In recent years, more attention has been devoted to impedance transducers with the capacity of label-free detection.

Bioaffinity based electrochemical biosensors are usually applied to detect gene mutations. A single stranded DNA sequence is immobilized on the electrode surface and when DNA hybridisation takes place, detection is conducted. A platform combined enzyme reaction with electrochemiluminescence (ECL) for sensitive detection of a single point mutation was developed. The electrode surface was coated by a composite of multiwalled carbon nanotubes and Ruthenium (II) tris-(bipyridine), and then covered by polypyrrole to immobilize ssDNA, which could recognize the gold nanoparticle (AuNP) labeled *p53* gene, and produce AuNP dsDNA electrode. Glucose dehydrogenase molecules were then absorbed for producing ECL signal. The system could recognize wild type *p53* sequences as low as 0.1 pM and the discrimination between wild type *p53* and mutation *p53* sequences was up to 56.3% [86]. Electrochemical sensors are also useful for detecting small damage to DNA. The electrochemical response of DNA is strongly dependent on the DNA structure [98]. Esterase 2-oligodeoxynucleotide conjugates were used for the electrochemical detection of mature microRNAs. The enzyme would be brought into the vicinity of the electrode and produces an electrochemical signal, when complementary binding of microRNA to the gap built of capture and detector oligodeoxynucleotide takes place. Without the target microRNA, the gap between capture and detector oligodeoxynucleotide was not filled, and missing base stacking energy destabilized the hybridization complex. Thus no signal was detected from the dissociative enzyme. The whole process took only 60 min and no reverse transcription PCR amplification was needed. A detection limit of 2 pM of *miR-16* within a mixture of other miRNAs was obtained [99].

Electrochemical detection of proteins is also widely used. There are two main detection principles. The antibody-antigen interaction could cause changes in the electrochemical properties (conductivity or capacitance) of the sensor surface. In the ELISA based assays, the reporter antibody or antigen is labeled with enzymes such as horseradish peroxidase (HRP), or alkaline phosphatase (AP), which will catalyze substrates to produce an electroactive species.

Magnetic particles were used in the elctrochemical biosensors for signal enhancement [100]. A gold interdigitated capacitor transducer was modified with magnetic beads (MB), and multiple detection of the protein biomarkers of lung cancer including carcinoembryonic antigen (CEA), epidermal growth factor receptor (hEGFR) and cancer antigen 15-3 (CA15-3) was investigated. CEA and hEGFR could be detected in the concentration range of 5 pg/mL to 1 ng/mL while CA15-3 was detected in the range of 1–200 U/mL with a high specificity [100]. Another sensor using magnetic nanoparticles Fe_3_O_4_@Au was constructed on one screen-printed carbon electrode (SPCE) for the rapid determination of highly sensitive C reactiveprotein (hs-CRP), and showed an excellent electrocatalytic activity for hs-CRP in PBS buffer, while a linear decrease of the catalytic efficiency of the HRP took place. The detection limit was 0.5 ng/mL and linear range was from 1.2 to 200 ng/mL in real serum samples. The accuracy and precision of the assay were 91.5%–104.4% and 15.8%–24.4% respectively [101]. The immunosensor is reusable once constructed and can be regenerated by adding new nanoprobes on the surface of basal electrode through magnet on its bottom. It can greatly reduce the detection cost which is valuable for screening of tumors.

#### 3.3.2. Mass Sensitive Transducer

Many physical and chemical processes are associated with mass changes. The piezoelectric quartz crystal resonator makes the microbalance possible. The application of an external electrical potential to a piezoelectric material produces internal methanical stress. The QCM is a piezoelectric sensor comprises a thin quartz crystal disk coated with gold electrode. An oscillating electric field is applied across the device, inducing an acoustic wave that propagates through the crystal and meets minimum pimpedance when the thickness of the device is a multiple of the half wavelength of the acoustic wave, so the QCM device is accurately a thickness shear mode resonator. The mass of a thin layer attached to the surface of a crystal can be calculated from a measured change in the resonant frequency of the device. The resonance frequency has been proved to decrease linearly with increasing mass on the QCM electrode at a nanogram level. QCM is a very sensitive mass-measuring device, and has been employed to observe real time biological events.

Using antibodies as the crystal coating, the QCM-based immunosensors could be used for protein marker detection. However, the QCM is associated with a lack of reproducibility in solution. To solve the problem, a “dip and dry” method was developed. Before the addition of samples, the resonance frequency of the crystal is determined in air. After the sample is dried, the change in resonant frequency due to biospecific binding could be calculated. QCM was used for the detection of one-point mutation of *TP53* gene. Amine coupling was employed for the immobilization of NeutrAvidin on the thiolderivatized surface. A 0.03–2 μM concentration range was detected, and there was no different between the SPR and QCM biosensors on the detection efficiency [102]. Chen Y. *et al.* applied the QCM immunosensor to detection early lung cancer rapidly. Murine Lewis lung carcinoma LL/2 cells were immobilized onto the surface of quartz crystals, and then the serum sample of LL/2 cell immunized rabbits was dripped onto the quartz crystal surface center. The additional mass of the crystal caused by specifically adsorbing antibodies resulted in a change in resonant frequency. The antibody content could be detected rapidly at the nanogram level with a high detection precision and a positive detection rate of above 80% [103]. With similar process, other cancer cells or antigens were detected using QCM immunosensor [104,105]. Sadhasivam S. *et al.* [105] reused up to nine cycles with a slight loss in binding affinity.

#### 3.3.3. Optical Transducers

##### 3.3.3.1. SPR

Optical transducers used in biosensors include fluorescence, interferometry and spectroscopy of optical waveguides, and surface plasmons resonance (SPR). SPR is one of the most common label-free optical techniques used for biomolecular interaction detection [106,107]. SPR biosensor is based on wavelength modulation and the Kretschmann geometry of the attenuated total reflection method. Briefly, a collimated polychromatic light beam from a halogen lamp passes through an optical prism and contacts a thin gold layer at a defined angle of incidence. Upon the incidence of the thin gold film, each light beam excited a surface plasmon at a certain wavelength. The reflected light is collected by an instrument with measured channels. Acquired spectra were analyzed in real time. The refractive index creates a characteristic dip in the detected light spectrum at a specific wavelength. Changes in refractive index cause this characteristic dip to shift. During the experiments a constant temperature is maintained to eliminate effects from temperature changes, so the changes are associated with the binding events occurring on the surface only, which could be quantified by tracking these changes in dip position [42].

For the early cancer biomarker detection, a glass chip is coated with a chromium or titanium film (thickness, 2 nm) and a gold film (thickness, 45–60 nm). The capture molecules are immobilized on the gold surface and unlabeled analyte is added. The refractive index changes at the interface of the gold sensor chip when biomolecules interact with immobilized capture agents, and is detected via angle changes of a reflected laser beam, which is directly related to the amount of sensor surface-bound biomolecules. The SPR sensor is a really versatile tool, but enables analysis of only a few channels in a single experiment. This method requires a large number of samples to be placed on the gold surfaces for microarray formats. Choi S. and Chae J. reported a novel SPR sensor using competitive protein adsorption to detect thyroglobulin instead of capture agents [108]. Two surfaces were covered by two known proteins with different affinities. Utilizing the competitive protein adsorption (IgG < Tg < fibrinogen), Tg displaced IgG, while Fibrinogen was not displaced, and Tg was selectively detected based on the exchange reaction.

SPR biosensors are sensitive to any refractive index change of the solution injected on the sensor surface. Refractive index mismatch of the buffer, the sample, as well as the running buffer can become a problem. Using a control serum solution that does not contain elevated levels of the antigen as running buffer is theoretically possible, but not feasible due to cost and health issues, while performing a sandwich assay instead of a direct assay will help in eliminating this problem. Cancer marker CA 19-9 was detected using SPR biosensor with or without a sandwich complex. The detection limit of CA19-9 was calculated to be 410.9 U/mL, while detection limit was improved to 66.7 U/mL with the antibodies against CA 19-9 additionally injected after each sample injection for the formation of sandwich complex [89]. A lot of studies have compared the SPR and ELISA methods. Vaisocherova H. *et al.* found that SPR detected CD166/activated cell leukocyte adhesion molecule (ALCAM), with a similar sensitivity to ELISA. Yet the SPR method took much less time [109]. The comparison was also put forward between SPR and QCM [110].

The main difficulty of testing real serum is the high nonspecific interaction between the sensor surface and serum proteins. Several strategies have been adopted to reduce the nonspecific binding, such as the use of mixed self-assembled monolayer coating which contain ethylene glycol units [111] and carboxymethyl dextran surface [112], additives in the assay buffer [113], blocking agents after antibody immobilization, and diluting the serum [111]. Nonspecific binding from 1% human serum influenced the standard curve profile, but did not influence the detection limit. This suggested that some of the antibody binding sites were covered with ligands bound from serum and were no longer accessible. When the serum concentration was increased to 10%, nonspecific binding was significantly increased and the detection limit was changed from 1 to 10 ng/mL [109]. To ultrasensitively detect the extremely small changes in refractive index, several experimental and theoretical approaches have been developed. For the detection of enzymes, surface modification of mixed self-assembled monolayers (SAMs) enhanced the SPR signal as a result of the reduction in steric hindrance [114]. The use of noble metal nanoparticles allowed strong optical coupling of incident light to resonances and localized surface plasmons (LSPs), which were collective electron oscillations localized in the metallic nanostructure [115]. Au nanoparticles are employed to enhance and amplify the sensor signal. A research investigated the effect of three gold nanoparticles with different size, on the conventional SPR signal, and found that the SPR signal was varied with the shapes and sizes of gold nanoparticles in suspension at a fixed concentration due to their different plasmon absorbance bands. Larger AuNPs not only occupy a larger space on the sensor surface that changes the refractive index, but also increase its participation to surface plasmon resonance as the volume of the Au, and hence enhance sensitivity further. The use of 20 nm Au nanoparticles reduced the detection limit 8 times, while the use of 40 nm Au nanoparticles lowed the detection limit 65 times. The signal enhancement correlates very well with the difference in their volume [110]. The composite material system of AuNPs and a SiO_2_ layer also successfully enhanced detection sensitivity by establishing the dielectric constants of the different component layers [116]. The detection limit of PSA by a SPR with the SiO_2_ layer and the AuNPs on the gold surface was 0.1 ng/mL, which was about 100 fold more sensitive than ELISA. SPR sensors were very stable and provided good reproducible responses after regeneration, up to 32 times [117].

##### 3.3.3.2. SPR Imaging (SPRi)

Recently, the SPR imaging (SPRi) technique has been at the forefront of optical label-free and real-time detection. SPRi systems are generally based on intensity modulation, measuring the reflectivity of monochromatic incident p-polarized light at a fixed angle. S-polarized light could also be measured, but is only used as reference signal to improve the image contrast and to eliminate artefacts. The sensing surface of the prism is coated with a thin gold or silver layer. The resonance conditions depend on the characteristics of the prism, metal and dielectric medium. The device can be used as a multichannel sensor when the sensor surface is divided into multiple sensing spots. SPRi could monitore hundreds of biological interactions continuously and simultaneously, and controlling the quality of the spotted array by selecting the measurement regions according to shape, size and quality [118]. A number of commercial SPRi instruments have recently been launched on the market. Three model biomarkers, α-fetoprotein, carcinoembryonic antigen, and hepatitis B surface antigen were simultaneously detected in human serum samples by a SPRi chip, showing detection limits of 50, 20, and 100 ng/mL, respectively [119]. SPRi and MALDI-TOF mass spectrometry were coupled in a hyphenated technique which enabled multiplexed quantification of binding by SPRi and molecular characterization of interacting partners by subsequent MS analysis [82].

##### 3.3.3.3. Fluorescence Biosensor

###### 3.3.3.3.1. Chromophore

Fluorescence techniques are very well suited to realizing the sensitive, reliable and reproducible detection of early cancer biomarkers, while fluorescence methods encompass several unique experimental parameters, for instance, excitation and emission wavelength, intensity, fluorescence lifetime and emission anisotropy, which are determined by the chromophore [120]. A variety of chromophores could be used, such as organic dyes, metal-ligand complexes, lanthanide chelates, and nanocrystals. Organic dyes are widely used for *in vitro* fluorescence quantification applications, for example, ELISA and real-time quantitative RT-PCR. The conjugation of organic dyes to biomolecules can be easily performed without significantly altering the biological functions. However, the spectral overlap problems of organic dyes need to be overcome for multiplexed approaches. Also, the fluorescence lifetime of organic dyes is too short for efficient discrimination [121]. Semiconductor quantum dots (QDs) show distinct advantages over organic dyes due to their spectroscopic properties: size-tunable absorption and emission, broad absorption, narrow and symmetric photoluminescence spectra, strong luminescence, robust photostability, and large surface-to-volume ratio [122–128]. Fluorescence quantum yields of QDs in the visible-NIR wavelength and the NIR wavelength are as high as those in the visible light range [129–134]. Resch-Genger U. *et al.* have compared the differences between organic dyes and QDs, and evaluated the advantages and limitations of both classes of chromophores [120].

###### 3.3.3.3.2. QDs as Chromophore

Luminescent semiconductor nanocrystals were utilized as fluorescent biological labels for the first time in 1998. Semiconductor quantum dots that covalently coupled to biomolecules, could recognize specific antibodies or antigens as fluorescent probes in biological staining and diagnostics [127,135]. The QDs that are synthesized in the organic phase possess better quality, but require additional post-treatment to gain water dispersibility [136,137]. In contrast, QDs prepared in the aqueous phase possess excellent aqueous dispersibility, while fluorescence quantum yields are lower than these synthesized in the organic phase. Highly luminescent QDs have been readily achieved in the aqueous phase through several strategies [137–140]. Our group presented the program process of microwave irradiation (PPMI), to synthesize high-quality CdTe nanocrystals with narrow size distribution and fewer surface defects in aqueous solution [141]. The method was consummated ulteriorly; CdTe nanocrystals with a PLQY of 82% were synthesized. Moreover, the PLQY increased to a remarkable 98% after further amelioration through the illumination method [142]. For the biological applications, CdTe/CdS/ZnS core/shell/shell (CSS) QDs with outstanding aqueous dispersibility, good spectral properties, excellent photostability, and favorable biocompatibility were synthesized [143]. Based on this work, a one-pot polymer encapsulation method was developed for group II-VI QDs in aqueous solution. The micelles of amphiphilic polymers captured and encapsulated the QDs, enhancing the photoluminescence quantum yield by about 50%, and so did the photostability [144]. Then we explored proline dithiocarbamic acid disodium salt (ProDTC) as a novel ligand for the first time, and made the synthesis of CdTe QDs progress at low temperature (30–50 °C). After replacing the surface-binding ProDTC molecules with mercaptopropionic acid, the photoluminescence quantum yield was about 50% [145]. All our work provides good conditions for studying nano-material fluorescence biosensors.

Besides the chemical nature and size and fluorophore properties of the QDs, the interplay between QDs and biomolecules would also affect the detection limit, the dynamic range, the reliability, and the suitability for multiplexing detection. Labeling of proteins or oliginucleotides with QDs requires suitable functional groups for covalent binding or noncovalent attachment. Aqueous QDs could bind to biomoleculars through electrostatical adsorption, coordinated compound, biotin-avidin interactions, covalent cross-linking (amine and carboxylic groups, amine and sulfhydryl groups, or aldehyde and hydrazide functions), or polyhistidine tags [146–151]. Our group developed a DNA-bridged strategy to facilely conjugate streptavidin (STV) to QDs, leading to a convenient and stable QD-DNA-biotin-STV conjugate, which served as fluorescent nanoprobes for ultrasensitive detection of cancer biomarkers [147]. These QD-based bioconjugates have been applied into ELISA, western blot, and microarray, and so on [152–154].

###### 3.3.3.3.3. Microfluidic Chip

In static solid/liquid interface reaction systems, QDs are less active than organic dyes because the QDs are prone to deposit on the solid surfaces. To overcome the problem, microfluidic chips based on the manipulation of a continuous liquid flow through microfabricated channels are applied, and we found that QDs were compatible with microfluidic chips [155]. The ambulatory fluid accelerates the reaction of QD probes and targets, and prevents QDs from nonspecific deposition [148]. These chips are highly sensitive for use in cancer biomarker assays. Our group developed a microfluidic protein chip with QDs as fluorescent signal amplifiers for ultrasensitive and multiplexed assay of cancer biomarkers, and it was selective enough to be used in sera directly. The microfluidic chip combines the high-throughput capabilities of a microfluidic network with the high sensitivity and multicolor imaging ability of QDs [148]. The QD-DNA-biotin-STV conjugates mentioned previously were also applied for ultrasensitive detection of cancer biomarkers within a microfluidic protein chip [147].

###### 3.3.3.3.4. Fluorescence Resonance Energy Transfer (FRET)

FRET, which has been reviewed previously [156–158], is a distance dependent energy transfer between a donor and an acceptor separated by a distance of 1–20 nm. As many biomolecular interactions occur in this distance range, FRET is frequently used for the parallel detection of several molecular interactions. The acceptor absorbs energy at the emission wavelength of the donor, but does not necessarily remit the energy fluorescently itself. The distance between the donor and acceptor molecules would affect the rate of energy transfer violently, making FRET very appealing for bioanalysis because of its intrinsic sensitivity to D/A distance [156]. Organic dyes and quenchers, polymers, metal chelates, QDs, and fluorescent proteins and amino acids, *etc.* can all function as either donors or acceptors. As mentioned previously, the unique photophysical properties of QDs make them attractive biolabels: high quantum yields, size-tunable photoluminescent emission, broad absorption spectra, large stokes shifts, multiphoton absorption, and high resistance to photobleaching and chemical degradation. QDs used as donors of FRET for identification of autoantibodies, tumor-specific T cells, and circulating cancer cells have been reviewed [159]. In this review, new materials used in FRET are highlighted. Our group used FRET from PFCOOH-BT(5) to diamines and biogenic polyamines to achieve a LOD as low as 2 μM, indicating that PFCOOH-BT(5) can serve as a general and effective chemosensor to detect trace diamines and biogenic polyamines [160].

Quenching acceptors become popular in FRET systems, because they could offer elimination of background fluorescence originating from direct acceptor excitation or re-emission. Recently, graphene oxide [161–169] and single-layer MoS_2_ [170] are used to quench fluorescence of organic dyes or QDs as quenching acceptors. GO and single-layer MoS_2_ could absorb chromophore-labeled ssDNA probe via the van der Waals force and then quench the fluorescence of chromophore. While the ssDNA is hybridized with its target DNA, RNA, ions, or proteins, the interaction becomes weak, and the chromophore would be away from the GO or MoS_2_ far enough to resume the fluorescence. A “turn-on” fluorescent biosensor is constructed. Zhu C.F. *et al.* used single-layer MoS_2_ to detect DNA [170]. Based on the interaction of the ssDNA aptamers and proteins, GO-based biosensor was developed for the tumor marker detection [167]. Tu Y.Q. *et al.* coupled the fluorescence quenching of GO with site-specific cleavage of *Rsa*I endonuclease. The biosensor could detected *miR-126* down to ~3.0 fM, and discriminated the target sequence from single-base mismatched sequence, and also estimated the *miR-126* expressions in cells [171]. Pei H. *et al.* reported a new concept of adaptive “ensemble aptamers” that exploited the collective recognition abilities of a set of aptamers to identify target discriminatively. GO could provide low background and highly reproducible fluorescent assay system [172]. Multiplexed detection of five diagnositic biomarkers at very low concentrations was realized through an optically multiplexed six-color FRET biosensor. With a sophisticated spectral crosstalk correction, the biosensor offered a nanogram level LOD for all five markers, providing an effective early screening tool for lung cancer. The technology opened a new door to multiple biomarker diagnostics [173]. Gold behaves like a dielectric with a large extinction index under blue or violet light. Presence of a transparent surface layer on gold produces a large decrease in the reflectivity of the gold surface due to multiple reflections in the surface layer, which is called anomalous reflection (AR) of the gold surface. The application of AR to real-time measurements of the adsorption process of octadecanethiol (ODT) on gold and the affinity of streptavidin to a biotin-labeled monomolecular layer on gold were denomstrated [174].

More sensitive and rapid technology platforms are needed to fulfill the diagnosis requirements in cancer detection, helping in providing better health care and reducing the stress on the patients. However, the readout of fluorescence biosensors is expensive and different for “point of care”, while label-free biosensors are capable of actualizing real-time, multi-analyte and continuous measurements. On the other hand, there are two disadvantages of these label-free biosensors, including limited sensitivity and the requirement of assay optimization and correct use of controls to differentiate nonspecific binding from specific especially using complex matrixes.

## 4. Conclusions

Early detection could help reduce the mortality rate of lung cancer. However, early detection is possible only through widespread screening, because of the asymptomatic onset of the disease. Associated with serum biomarkers, the development of biosensors provides an inexpensive, easy to use, portable, non-invasive tool with high sensitivity and specificity, showing great potential for routine diagnostics. The available biomarkers still lack sufficient specificity and sensitivity for use in early cancer diagnosis [43]. Hence, identification of such biomarkers as occur at an early stage during tumorigenesis will further improve. On the other hand, there is a lack of standard of biomarkers for cancer diagnosis. Firstly, the specificity of biomarkers corresponding to certain cancer is not affirmatory [175]. Secondly, the inhomogeneity of cancer results in the inhomogeneity of biomarkers, which makes the diagnosis intricate. Fortunately, the combination of biomarkers used for early cancer detection was proposed. The threshold concentration of biomarkers signifying cancer also needs to be set [176]. Biosensors have the potential to detect cancer biomarkers sensitively, specifically and simply. However, biosensor devices need further developmnt to face the multiplex analysis, small sample consumption, reduced assay time, low manufacturing cost, and high throughput, along with improved diagnostic accuracy as well as repeatability. The concept of using nanomaterials and microfluidics will make biosensors more sensitive and more applicable for high-throughput assay. So-called “point of care” biosensors will help reduce the mortality rate of lung cancer by early diagnosis and prognosis.

## Figures and Tables

**Table 1:** Tumor-associated antigens (TAA) panels used for lung cancer diagnosis. SqLCC, Squamous Lung Cell Carcinoma; NSCLC, Non-Small Cell Lung Cancer; SCLC, Small Cell Lung Carcinoma.

The panel of TAAs	Cancer type	Sensitivity (%)	Specificity (%)	Ref.
14-2-2 theta, Annexin I, PGP 9.5	Lung cancer	55	95	[47]
Annexin I, 14-3-3 theta, LAMR1	Lung cancer	-	-	[67]
Alpha-enolase, Carcinoembryonic antigen, Cytokeratin 19 fragment (CYFRA21-1)	NSCLC	-	-	[69]
C-myc, p16, p53	Lung cancer	17.9	-	[76]
TP53 BP, Protein kinase C and Lymphoid blast crisis oncogene (LBC)	SqLCC	-	-	[77]
SOX1, SOX2, SOX3, SOX21, HuC, HuD, or HeIN1 proteins	SCLC	67	95	[78,79]
Six phage peptides	NSCLC	92	85	[51]
HER2, p53, c-myc, NY-ESO-1, CAGE, MUC1 and GBU4-5	NSCLC and SCLC	64–92	92–100	[75]
Six phage peptides	NSCLC	94.8	91.1	[7]

**Table 2:** The limit of detection (LOD) and linear range of the lung cancer biomarker detection using biosensors. VEGF, vascular endothelial growth factor; CEA, carcinoembryo antigen; COX, cyclooxygenase; ALCAM, activated leukocyte cell adhesion molecule; EGFR, epidermal growth factor receptor; TAGLN2, transgelin-2; hCG, human chorionic gonadotropin; PNA, peptide nucleic acid.

Biomarker	Capture agent	Sample	Transducer	LOD	Linear range	Ref.
VEGF	VEG-Freceptor-1	Serum	Electrochemical	-	10–70 pg/mL	[74]
	Aptamer	-	Electrochemical	15 nM	-	[80]
VEGF165	Aptamer	Serum	Fluorescent	-	1.25 pM to 1.25 μM	[81]
LAG3 protein	Antibody	Plasma	SPRi-MALDI-TOP MS	-	-	[82]
*TP53* gene	DNA	-	Optical (SPR) and QCM	-	0.3–2 μM	[83]
COX-2	Polyclona antibody	Simulated blood sample	Optical (SPR)	1.35 × 10^−4^ ng/mL	3.64 × 10^−4^–3.64 × 10^2^ ng/mL	[84]
			Fluorescence	1.02 × 10^−4^ ng/mL	7.46 × 10^−4^–7.46 × 10^1^ ng/mL	
CEA	Antibody	Serum	Optical (SPR)	-	-	[42]
p53 antibody	p53 antigen	Serum	Microcantilever biosensor	-	20 ng/mL–20 μg/mL	[85]
*p53*	ssDNA	-	Electrochemilumi nescence	-	-	[86]
*p53* (wild and total)	ds-DNA and antibody	-	Optical (SPR)	10.6 and 1.06 pM	-	[87]
EGFR	Aptamer	Serum	Optical	-	-	[88]
CA 19-9	Antibody	-	Optical (SPR)	66.7 U/mL	-	[89]
ALCAM	Antibody	10% Serum	Optical (SPRi)	6 ng/mL	-	[90]
ALCAM and hCG	antibody	10% Serum	Optical (SPRi)	45–100 ng/mL	-	[91]
TAGLN2	Antibody	10% Serum	Optical (SPRi)	3 ng/mL	-	[90]
DNA mutations	ssDNA	Serum	Optical (SPR)	50 nM	-	[92]
K-*ras* point mutation	PNA	-	Optical (SPR)	-	-	[93]

## References

[1] Jemal A., Bray F., Center M.M., Ferlay J., Ward E., Forman D. (2011). Global cancer statistics. CA: Cancer J. Clin.

[2] Ferlay J., Shin H.-R., Bray F., Forman D., Mathers C., Parkin D.M. (2010). Estimates of worldwide burden of cancer in 2008: Globocan 2008. Int. J. Cancer.

[3] Chiang C.J., Chen Y.C., Chen C.J., You S.L., Lai M.S. (2010). Cancer trends in Taiwan. Jpn. J. Clin. Oncol.

[4] Bray F., Jemal A., Grey N., Ferlay J., Forman D. (2012). Global cancer transitions according to the human development index (2008–2030): A population-based study. Lancet Oncol.

[5] Reddy C., Chilla D., Boltax J. (2011). Lung cancer screening: A review of available data and current guidelines. Hosp. Pract.

[6] Shirvani S.M., Jiang J., Chang J.Y., Welsh J.W., Gomez D.R., Swisher S., Buchholz T.A., Smith B.D. (2012). Comparative effectiveness of 5 treatment strategies for early-stage non-small cell lung cancer in the elderly. Int. J. of Radiat. Oncol. Biol. Phys.

[7] Farlow E.C., Patel K., Basu S., Lee B.S., Kim A.W., Coon J.S., Faber L.P., Bonomi P., Liptay M.J., Borgia J.A. (2010). Development of a multiplexed tumor-associated autoantibody-based blood test for the detection of non-small cell lung cancer. Clin. Cancer Res.

[8] Conrad D.H., Goyette J., Thomas P.S. (2008). Proteomics as a Method for early detection of cancer: A review of proteomics, exhaled breath condensate, and lung cancer screening. J. Gen. Intern. Med.

[9] Aberle D.R., Adams A.M., Berg C.D., Black W.C., Clapp J.D., Fagerstrom R.M., Gareen I.F., Gatsonis C., Marcus P.M., Sicks J.D. (2011). Reduced lung-cancer mortality with low-dose computed tomographic screening. N. Engl. J. Med.

[10] Oken M.M., Hocking W.G., Kvale P.A., Andriole G.L., Buys S.S., Church T.R., Crawford E.D., Fouad M.N., Lsaacs C., Reding D.J. (2011). Screening by chest radiograph and lung cancer mortality the prostate, lung, colorectal, and ovarian (plco) randomized trial. J. Am. Med. Assoc.

[11] Smith R.A., Brooks D., Cokkinides V., Saslow D., Brawley O.W. (2013). Cancer screening in the united states, 2013: A review of current american cancer society guidelines, current issues in cancer screening, and new guidance on cervical cancer screening and lung cancer screening. CA: Cancer J. Clin.

[12] Chicklore S., Goh V., Siddique M., Roy A., Marsden P.K., Cook G.J.R. (2013). Quantifying Tumour Heterogeneity in F-18-Fdg Pet/Ct Imaging by Texture Analysis. Eur. J. Nucl. Med. Mol. Imaging.

[13] Asselin M.C., O’Connor J.P.B., Boellaard R., Thacker N.A., Jackson A. (2012). Quantifying heterogeneity in human tumours using Mri and Pet. Eur. J. Cancer.

[14] De Geus-Oei L.-F., van der Heijden H.F.M., Visser E.P., Hermsen R., van Hoom B.A., Timmer-Bonte J.N.H., Willemsen A.T., Pruim J., Corstens F.H.M., Krabbe P.F.M. (2007). Chemotherapy response evaluation with f-18-fdg pet in patients with non-small cell lung cancer. J. Nucl. Med.

[15] Ippolito D., Capraro C., Guerra L., De Ponti E., Messa C., Sironi S. (2013). Feasibility of perfusion ct technique integrated into conventional (18)fdg/pet-ct studies in lung cancer patients: Clinical staging and functional information in a single study. Eur. J. Nucl. Med. Mol. Imaging.

[16] Peng G., Hakim M., Broza Y.Y., Billan S., Abdah-Bortnyak R., Kuten A., Tisch U., Haick H. (2010). Detection of lung, breast, colorectal, and prostate cancers from exhaled breath using a single array of nanosensors. Br. J. Cancer.

[17] Balgkouranidou I., Liloglou T., Lianidou E.S. (2013). Lung cancer epigenetics: emerging biomarkers. Biomark. Med.

[18] Hassanein M., Callison J.C., Callaway-Lane C., Aldrich M.C., Grogan E.L., Massion P.P. (2012). The state of molecular biomarkers for the early detection of lung cancer. Cancer Prev. Res.

[19] Zhong L., Coe S.P., Stromberg A.J., Khattar N.H., Jett J.R., Hirschowitz E.A. (2006). Profiling tumor-associated antibodies for early detection of non-small cell lung cancer. J. Thorac. Oncol.

[20] Tothill I.E. (2009). Biosensors for cancer markers diagnosis. Semin. Cell Dev. Biol.

[21] Tomizaki K.-Y., Usui K., Mihara H. (2005). Protein-detecting microarrays: current accomplishments and requirements. ChemBioChem.

[22] Nowsheen S., Aziz K., Panayiotidis M.I., Georgakilas A.G. (2012). Molecular markers for cancer prognosis and treatment: Have we struck gold?. Cancer Lett.

[23] Calin G.A., Croce C.M. (2006). Microrna signatures in human cancers. Nat. Reviews Cancer.

[24] Esquela-Kerscher A., Slack F.J. (2006). Oncomirs-micrornas with a role in Cancer. Nat. Rev. Cancer.

[25] Chen X., Ba Y., Ma L., Cai X., Yin Y., Wang K., Guo J., Zhang Y., Chen J., Guo X. (2008). Characterization of micrornas in serum: A novel class of biomarkers for diagnosis of cancer and other diseases. Cell Res.

[26] Lodes M.J., Caraballo M., Suciu D., Munro S., Kumar A., Anderson B. (2009). Detection of cancer with serum mirnas on an oligonucleotide microarray. PLoS One.

[27] Hu Z., Yang Z., Tian T., Liang J., Jin G., Shen H. (2009). Association between microrna polymorphisms, expressions, lung cancer development and prognosis. Biomed. Pharmacother.

[28] Yanaihara N., Caplen N., Bowman E., Seike M., Kumamoto K., Yi M., Stephens R.M., Okamoto A., Yokota J., Tanaka T. (2006). Unique microrna molecular profiles in lung cancer diagnosis and prognosis. Cancer Cell.

[29] Zandberga E., Kozirovskis V., Abols A., Andrejeva D., Purkalne G., Line A. (2013). Cell-free micrornas as diagnostic, prognostic, and predictive biomarkers for lung cancer. Genes Chromosomes Cancer.

[30] Schmitt M.J., Margue C., Behrmann I., Kreis S. (2013). *Mirna-29*: A microrna family with tumor-suppressing and immune-modulating properties. Curr. Mol. Med.

[31] Wu J.J., Yang T., Li X., Yang Q.Y., Liu R., Huang J.K., Li Y.Q., Yang C.F., Jiang Y.G. (2013). Alteration of serum *mir-206* and *mir-133b* is associated with lung carcinogenesis induced by 4-(methylnitrosamino)-1-(3-pyridyl)-1-butanone. Toxicol. Appl. Pharmacol.

[32] Miao L.J., Huang S.F., Sun Z.T., Gao Z.Y., Zhang R.X., Liu Y., Wang J. (2013). *Mir-449c* targets C-myc and inhibits nsclc cell progression. FEBS Lett.

[33] Kaduthanam S., Gade S., Meister M., Brase J.C., Johannes M., Dienemann H., Warth A., Schnabel P.A., Herth F.J.F., Sultmann H. (2013). Serum *mir-142–3p* is associated with early relapse in operable lung adenocarcinoma patients. Lung Cancer (Amsterdam, The Netherlands).

[34] Huang B., Luo W., Sun L., Zhang Q., Jiang L., Chang J., Qiu X., Wang E. (2013). *Mirna-125a-3p* is a negative regulator of the rhoa-actomyosin pathway in a549 cells. Int. J. Oncol.

[35] Lubin R., Zalcman G., Bouchet L., Tredaniel J., Legros Y., Cazals D., Hirsch A., Soussi T. (1995). Serum P53 antibodies as early markers of lung-cancer. Nat. Med.

[36] Trivers G.E., DeBenedetti V.M.G., Gawley H.L., Caron G., Harrington A.M., Bennett W.P., Jett J.R., Colby T.V., Tazelaar H., Pairolero P. (1996). Anti-p53 antibodies in sera from patients with chronic obstructive pulmonary disease can predate a diagnosis of cancer. Clin. Cancer Res.

[37] Bermejo-Perez M.J., Marquez-Calderon S., Lianos-Mendez A. (2008). Cancer Surveillance based on imaging techniques in carriers of brca1/2 gene mutations: A Systematic review. Br. J. Radiol.

[38] Chapman C., Murray A., Chakrabarti J., Thorpe A., Woolston C., Sahin U., Barnes A., Robertson J. (2007). Autoantibodies in breast cancer: Their use as an aid to early diagnosis. Ann. Oncol.

[39] Luna Coronell J.A., Syed P., Sergelen K., Gyurjan I., Weinhausel A. (2012). The current status of cancer biomarker research using tumour-associated antigens for minimal invasive and early cancer diagnostics. J. Proteomics.

[40] Casiano C.A., Mediavilla-Varela M., Tan E.M. (2006). Tumor-associated antigen arrays for the serological diagnosis of cancer. Mol. Cell. Proteomics.

[41] Wagner P.D., Verma M., Srivastava S., Hoon D.S.B., Taback B. (2004). Challenges for biomarkers in cancer detection. Circulating Nucleic Acids in Plasma/Serum III and Serum Proteomics.

[42] Ladd J., Lu H., Taylor A.D., Goodell V., Disis M.L., Jiang S. (2009). Direct detection of carcinoembryonic antigen autoantibodies in clinical human serum samples using a surface plasmon resonance sensor. Coll. Surf. B.

[43] Tan H.T., Low J., Lim S.G., Chung M.C.M. (2009). Serum autoantibodies as biomarkers for early cancer detection. FEBS J.

[44] Gunawardana C.G., Diamandis E.P. (2007). High throughput proteomic strategies for identifying tumour-associated antigens. Cancer Lett.

[45] Brichory F., Beer D., Le Naour F., Giordano T., Hanash S. (2001). Proteomics-based identification of protein gene product 9.5 as a tumor antigen that induces a humoral immune response in lung cancer. Cancer Res.

[46] Brichory F.M., Misek D.E., Yim A.M., Krause M.C., Giordano T.J., Beer D.G., Hanash S.M. (2001). An immune response manifested by the common occurrence of annexins i and ii autoantibodies and high circulating levels of il-6 in lung cancer. Proc. Natl. Acad. Sci. USA.

[47] Pereira-Faca S.R., Kuick R., Puravs E., Zhang Q., Krasnoselsky A.L., Phanstiel D., Qiu J., Misek D.E., Hinderer R., Tammemagi M. (2007). Identification of 14-3-3 theta as an antigen that induces a humoral response in lung cancer. Cancer Res.

[48] Canelle L., Bousquet J., Pionneau C., Deneux L., Imam-Sghiouar N., Caron M., Joubert-Caron R. (2005). An efficient proteomics-based approach for the screening of autoantibodies. J. Immunol. Methods.

[49] Li C., Li X.Y., Tang C.E., Yi H., Duan C.J. (2006). Screen biomarkers of human lung squamous carcinoma by serological proteome analysis of htb-182. Transact. Nonferr. Met. Soc. China.

[50] Wang X.J., Yu J.J., Sreekumar A., Varambally S., Shen R.L., Giacherio D., Mehra R., Montie J.E., Pienta K.J., Sanda M.G. (2005). Autoantibody signatures in prostate cancer. N. Engl. J. Med.

[51] Wu L., Chang W., Zhao J., Yu Y., Tan X., Su T., Zhao L., Huang S., Liu S., Cao G. (2010). Development of autoantibody signatures as novel diagnostic biomarkers of non-small cell lung cancer. Clin. Cancer Res.

[52] Sahin U., Tureci O., Schmitt H., Cochlovius B., Johannes T., Schmits R., Stenner F., Luo G.R., Schobert I., Pfreundschuh M. (1995). Human neoplasms elicit multiple specific immune-responses in the autologous host. Proc. Natl. Acad. Sci. USA.

[53] Song M.H., Ha J.M., Shin D.H., Lee C.H., Old L., Lee S.Y. (2012). Kp-Cot-23 (Ccdc83) is a novel immunogenic cancer/testis antigen in colon cancer. Int. J. Oncol.

[54] Cancer Immunome Datebase (CID) Home Page.

[55] Liu H.Y., Peng L.P., Ran Y.L., Zhang L.Z., Zhang D.C., Zhu Y.K., Yang Z.H. (2002). Screening and identification of human lung cancer-related antigens. Acta Biochim. Et Biophys. Sinica.

[56] Gure A.O., Stockert E., Scanlan M.J., Keresztes R.S., Jager D., Altorki N.K., Old L.J., Chen Y.T. (2000). Serological identification of embryonic neural proteins as highly immunogenic tumor antigens in small cell lung cancer. Proc. Natl. Acad. Sci. USA.

[57] Kijanka G., Murphy D. (2009). Protein arrays as tools for serum autoantibody marker discovery in cancer. J. Proteomics.

[58] Nam M.J., Madoz-Gurpide J., Wang H., Lescure P., Schmalbach C.E., Zhao R., Misek D.E., Kuick R., Brenner D.E., Hanash S.M. (2003). Molecular profiling of the immune response in colon cancer using protein microarrays: Occurrence of autoantibodies to ubiquitin *C*-terminal hydrolase L3. Proteomics.

[59] Qiu J., Madoz-Gurpide J., Misek D.E., Kuick R., Brenner D.E., Michailidis G., Haab B.B., Omenn G.S., Hanash S. (2004). Development of natural protein microarrays for diagnosing cancer based on an antibody response to tumor antigens. J. Proteome Res.

[60] Madoz-Gurpide J., Wang H., Misek D.E., Brichory F., Hanash S.M. (2001). Protein based microarrays: A tool for probing the proteome of cancer cells and tissues. Proteomics.

[61] Qin S.Z., Qiu W.L., Ehrlich J.R., Ferdinand A.S., Richie J.P., O’Leary M.P., Lee M.L.T., Liu B.C.S. (2006). Development of a “Reverse Capture” autoantibody microarray for studies of antigen-autoantibody profiling. Proteomics.

[62] Ehrlich J.R., Tang L., Caiazzo R.J., Cramer D.W., Ng S.K., Ng S.W., Liu B.C.S. (2008). The “Reverse Capture” autoantibody microarray: An innovative approach to profiling the autoantibody response to tissue-derived native antigens. Methods Mol. Biol..

[63] Ehrlich J.R., Qin S., Liu B.C.S. (2006). The “Reverse Capture” autoantibody microarray: A native antigen-based platform for autoantibody profiling. Nat. Protoc.

[64] Miller J.C., Zhou H.P., Kwekel J., Cavallo R., Burke J., Butler E.B., Teh B.S., Haab B.B. (2003). Antibody Microarray profiling of human prostate cancer sera: Antibody screening and identification of potential biomarkers. Proteomics.

[65] Hardouin J., Lasserre J.-P., Sylvius L., Joubert-Caron R., Caron M. (2007). Cancer immunomics— From Serological proteome analysis to multiple affinity protein profiling. Ann. N. Y. Acad. Sci.

[66] Caron M., Joubert-Caron R., Canelle L., Hardouin J. (2005). Serological proteome analysis (serpa) and multiple affinity protein profiling (mapping) to discover cancer biomarkers. Mol. Cell. Proteomics.

[67] Qiu J., Choi G., Li L., Wang H., Pitteri S.J., Pereira-Faca S.R., Krasnoselsky A.L., Randolph T.W., Omenn G.S., Edelstein C. (2008). Occurrence of autoantibodies to annexin i, 14-3-3 theta and lamr1 in prediagnostic lung cancer sera. J. Clin. Oncol.

[68] Nakanishi T., Takeuchi T., Ueda K., Murao H., Shimizu A. (2006). Detection of eight antibodies in cancer patients’ sera against proteins derived from the adenocarcinoma a549 cell line using proteomics-based analysis. J. Chromatogr. B.

[69] He P., Naka T., Serada S., Fujimoto M., Tanaka T., Hashimoto S., Shima Y., Yamadori T., Suzuki H., Hirashima T. (2007). Proteomics-Based identification of alpha-enolase as a tumor antigen in non-small lung cancer. Cancer Sci.

[70] Yang F., Xiao Z.Q., Zhang X.Z., Li C., Zhang P.F., Li M.Y., Chen Y., Zhu G.Q., Sun Y., Liu Y.F. (2007). Identification of tumor antigens in human lung squamous carcinoma by serological proteome analysis. J. Proteome Res.

[71] Soussi T. (2000). P53 antibodies in the sera of patients with various types of cancer: A review. Cancer Res.

[72] Nagashio R., Sato Y., Jiang S.X., Ryuge S., Kodera Y., Maeda T., Nakajima T. (2008). Detection of tumor-specific autoantibodies in sera of patients with lung cancer. Lung Cancer.

[73] Tureci O., Mack U., Luxemburger U., Heinen H., Krummenauer F., Sester M., Sester U., Sybrecht G.W., Sahin U. (2006). Humoral immune responses of lung cancer patients against tumor antigen ny-eso-1. Cancer Lett.

[74] Sezginturk M.K. (2011). A new impedimetric biosensor utilizing vegf receptor-1 (flt-1): Early diagnosis of vascular endothelial growth factor in breast cancer. Biosens. Bioelectron.

[75] Chapman C.J., Murray A., McElveen J.E., Sahin U., Luxemburger U., Tuereci O., Wiewrodt R., Barnes A.C., Robertson J.F. (2008). Autoantibodies in lung cancer: Possibilities for early detection and subsequent cure. Thorax.

[76] Looi K., Megliorino R., Shi F.D., Peng X.X., Chen Y., Zhang J.Y. (2006). Humoral immune response to p16, a cyclin-dependent kinase inhibitor in human malignancies. Oncol. Rep.

[77] Diesinger I., Bauer C., Brass N., Schaefers H.J., Comtesse N., Sybrecht G., Meese E. (2002). Toward a more complete recognition of immunoreactive antigens in squamous cell lung carcinoma. Int. J. Cancer.

[78] Titulaer M.J., Klooster R., Potman M., Sabater L., Graus G., Hegeman I.M., Thijssen P.E., Wirtz P.W., Twijnstra A., Smitt P.A.E.S. (2009). Sox antibodies in small-cell lung cancer and lambert-eaton myasthenic syndrome: frequency and relation with survival. J. Clin. Oncol.

[79] Sabater L., Titulaer M., Saiz A., Verschuuren J., Guere A.O., Graus F. (2008). Sox1 antibodies are markers of paraneoplastic lambert-eaton myasthenic syndrome. Neurology.

[80] Nonaka Y., Abe K., Ikebukuro K. (2012). Electrochemical detection of vascular endothelial growth factor with aptamer sandwich. Electrochemistry.

[81] Cho H., Yeh E.C., Sinha R., Laurence T.A., Bearinger J.P., Lee L.P. (2012). Single-step nanoplasmonic vegf(165) aptasensor for early cancer diagnosis. ACS Nano.

[82] Remy-Martin F., El Osta M., Lucchi G., Zeggari R., Leblois T., Bellon S., Ducoroy P., Boireau W. (2012). Surface plasmon resonance imaging in arrays coupled with mass spectrometry (supra-ms): Proof of concept of on-chip characterization of a potential breast cancer marker in human plasma. Anal. Bioanal. Chem.

[83] Altintas Z., Uludag Y., Gurbuz Y., Tothill I.E. (2011). Surface plasmon resonance based immunosensor for the detection of the cancer biomarker carcinoembryonic antigen. Talanta.

[84] Noah N.M., Mwilu S.K., Sadik O.A., Fatah A.A., Arcilesi R.D. (2011). Immunosensors for quantifying cyclooxygenase 2 pain biomarkers. Clin. Chim. Acta.

[85] Zhou Y., Wang Z., Yue W., Tang K., Ruan W., Zhang Q., Liu L. (2009). Label-Free detection of p53 antibody using a microcantilever biosensor with piezoresistive readout. IEEE Sens.

[86] Wang X., Zhang X., He P., Fang Y. (2011). Sensitive detection of p53 tumor suppressor gene using an enzyme-based solid-state electrochemiluminescence sensing platform. Biosens. Bioelectron.

[87] Wang Y., Zhu X., Wu M., Xia N., Wang J., Zhou F. (2009). Simultaneous and label-free determination of wild-type and mutant p53 at a single surface plasmon resonance chip preimmobilized with consensus dna and monoclonal antibody. Anal. Chem.

[88] Ilyas A., Asghar W., Allen P.B., Duhon H., Ellington A.D., Iqbal S.M. (2012). Electrical detection of cancer biomarker using aptamers with nanogap break-junctions. Nanotechnology.

[89] Chung J.W., Bernhardt R., Pyun J.C. (2006). Additive assay of cancer marker ca 19–9 by spr biosensor. Sens. Actuators B.

[90] Ladd J., Taylor A.D., Piliarik M., Homola J., Jiang S. (2009). Label-free detection of cancer biomarker candidates using surface plasmon resonance imaging. Anal. Bioanal. Chem.

[91] Piliarik M., Bockova M., Homola J. (2010). Surface plasmon resonance biosensor for parallelized detection of protein biomarkers in diluted blood plasma. Biosens. Bioelectron.

[92] Carrascosa L.G., Calle A., Lechuga L.M. (2009). Label-free detection of dna mutations by spr: Application to the early detection of inherited breast cancer. Anal. Bioanal. Chem.

[93] Sato Y., Fujimoto K., Kawaguchi H. (2003). Detection of a k-ras point mutation employing peptide nucleic acid at the surface of a spr biosensor. Coll. Surf. B.

[94] McCauley T.G., Hamaguchi N., Stanton M. (2003). Aptamer-based biosensor arrays for detection and quantification of biological macromolecules. Anal. Biochem.

[95] Pan C., Guo M., Nie Z., Xiao X., Yao S. (2009). Aptamer-based electrochemical sensor for label-free recognition and detection of cancer cells. Electroanalysis.

[96] Glokler J., Angenendt P. (2003). Protein and antibody microarray technology. J. Chromatogr. B.

[97] Lin J.H., Ju H.X. (2005). Electrochemical and chemiluminescent immunosensors for tumor markers. Biosens. Bioelectron.

[98] Wang J. (2006). Electrochemical biosensors: Towards point-of-care cancer diagnostics. Biosens. Bioelectron.

[99] Pohlmann C., Sprinzl M. (2010). Electrochemical detection of micrornas via gap hybridization assay. Anal. Chem.

[100] Altintas Z., Kallempudi S.S., Sezerman U., Gurbuz Y. (2012). A novel magnetic particle-modified electrochemical sensor for immunosensor applications. Sens. Actuators B.

[101] Gan N., Meng L., Hu F., Cao Y., Wu Y., Jia L., Zheng L., Fan W. (2012). A renewable amperometric immunosensor for hs-crp based on functionalized Fe_3_O_4_@Au magnetic nanoparticles attracted on fe (iii) phthlocyanine/chitosan-membrane modified screen-printed carbon electrode by a magnet. Mechanical and Aerospace Engineering.

[102] Altintas Z., Tothill I.E. (2012). DNA-based biosensor platforms for the detection of tp53 mutation. Sens. Actuators B.

[103] Chen Y., Huang X., Shi H., Mu B. (2011). A novel and cost-effective method for early lung cancer detection in immunized serum. Asian Pac. J. Cancer Prev.

[104] Chen Y., Huang X.H., Shi H.S., Mu B., Lv Q. (2012). Rapid detection of ovarian cancer from immunized serum using a quartz crystal microbalance immunosensor. Asian Pac. J. Cancer Prev.

[105] Sadhasivam S., Chen J.C., Savitha S., Lin F.H., Yang Y.Y., Lee C.H. (2012). A real time detection of the ovarian tumor associated antigen 1 (ovta 1) in human serum by quartz crystal microbalance immobilized with anti-ovta 1 polyclonal chicken igy antibodies. Mater. Sci. Eng. C.

[106] Piliarik M., Vaisocherova H., Homola J. (2009). Surface plasmon resonance biosensing. Methods Mol. Biol.

[107] Donzella V., Crea F. (2011). Optical biosensors to analyze novel biomarkers in oncology. J. Biophotonics.

[108] Choi S., Chae J. (2009). A microfluidic biosensor based on competitive protein adsorption for thyroglobulin detection. Biosens. Bioelectron.

[109] Vaisocherova H., Faca V.M., Taylor A.D., Hanash S., Jiang S. (2009). Comparative study of spr and elisa methods based on analysis of cd166/alcam levels in cancer and control human sera. Biosens. Bioelectron.

[110] Uludag Y., Tothill I.E. (2012). Cancer biomarker detection in serum samples using surface plasmon resonance and quartz crystal microbalance sensors with nanoparticle signal amplification. Anal. Chem..

[111] Ayela C., Roquet F., Valera L., Granier C., Nicu L., Pugniere M. (2007). Antibody-antigenic peptide interactions monitored by spr and qcm-d-a model for spr detection of ia-2 autoantibodies in human serum. Biosens. Bioelectron.

[112] Situ C., Wylie A.R.G., Douglas A., Elliott C.T. (2008). Reduction of severe bovine serum associated matrix effects on carboxymethylated dextran coated biosensor surfaces. Talanta.

[113] Trevino J., Calle A., Rodriguez-Frade J.M., Mellado M., Lechuga L.M. (2009). Determination of human growth hormone in human serum samples by surface plasmon resonance immunoassay. Talanta.

[114] Choi S.H., Lee J.W., Sim S.J. (2005). Enhanced performance of a surface plasmon resonance immunosensor for detecting ab-gad antibody based on the modified self-assembled monolayers. Biosens. Bioelectron.

[115] Sato Y., Hosokawa K., Maeda M. (2008). Detection of non-cross-linking interaction between DNA-modified gold nanoparticles and a dna-modified flat gold surface using surface plasmon resonance imaging on a microchip. Coll. Surf. B.

[116] Jung J., Na K., Lee J., Kim K.W., Hyun J. (2009). Enhanced surface plasmon resonance by au nanoparticles immobilized on a dielectric sio2 layer on a gold surface. Anal. Chim. Acta.

[117] Suwansa-ard S., Kanatharana P., Asawatreratanakul P., Wongkittisuksa B., Limsakul C., Thavarungkul P. (2009). Comparison of surface plasmon resonance and capacitive immunosensors for cancer antigen 125 detection in human serum samples. Biosens. Bioelectron.

[118] Scarano S., Mascini M., Turner A.P.F., Minunni M. (2010). Surface plasmon resonance imaging for affinity-based biosensors. Biosens. Bioelectron.

[119] Hu W., Liu Y., Lu Z., Li C.M. (2010). Poly oligo(ethylene glycol) methacrylate-co-glycidyl methacrylate brush substrate for sensitive surface plasmon resonance imaging protein arrays. Adv. Funct. Mater.

[120] Resch-Genger U., Grabolle M., Cavaliere-Jaricot S., Nitschke R., Nann T. (2008). Quantum dots *versus* organic dyes as fluorescent labels. Nat. Methods.

[121] Mihindukulasuriya S.H., Morcone T.K., McGown L.B. (2003). Characterization of acridone dyes for use in four-decay detection in dna sequencing. Electrophoresis.

[122] Carion O., Mahler B., Pons T., Dubertret B. (2007). Synthesis, encapsulation, purification and coupling of single quantum dots in phospholipid micelles for their use in cellular and *in vivo* imaging. Nat. Protoc.

[123] So M.K., Xu C.J., Loening A.M., Gambhir S.S., Rao J.H. (2006). Self-illuminating quantum dot conjugates for *in vivo* imaging. Nat. Biotechnol.

[124] Wu X.Y., Liu H.J., Liu J.Q., Haley K.N., Treadway J.A., Larson J.P., Ge N.F., Peale F., Bruchez M.P. (2003). Immunofluorescent labeling of cancer marker her2 and other cellular targets with semiconductor quantum dots. Nat. Biotechnol.

[125] Gao X.H., Cui Y.Y., Levenson R.M., Chung L.W.K., Nie S.M. (2004). *In vivo* cancer targeting and imaging with semiconductor quantum dots. Nat. Biotechnol.

[126] Jaiswal J.K., Mattoussi H., Mauro J.M., Simon S.M. (2003). Long-term multiple color imaging of live cells using quantum dot bioconjugates. Nat. Biotechnol.

[127] Bruchez M., Moronne M., Gin P., Weiss S., Alivisatos A.P. (1998). Semiconductor nanocrystals as fluorescent biological labels. Science.

[128] Liu W., Howarth M., Greytak A.B., Zheng Y., Nocera D.G., Ting A.Y., Bawendi M.G. (2008). Compact biocompatible quantum dots functionalized for cellular imaging. J. Am. Chem. Soc.

[129] Hinds S., Myrskog S., Levina L., Koleilat G., Yang J., Kelley S.O., Sargent E.H. (2007). Nir-emitting colloidal quantum dots having 26% luminescence quantum yield in buffer solution. J. Am. Chem. Soc.

[130] Fernee M.J., Jensen P., Rubinsztein-Dunlop H. (2007). Origin of the large homogeneous line widths obtained from strongly quantum confined pbs nanocrystals at room temperature. J. Phys. Chem. C.

[131] Shavel A., Gaponik N., Eychmueller A. (2006). Factors governing the quality of aqueous cdte nanocrystals: calculations and experiment. J. Phys. Chem. B.

[132] Jiang W., Singhal A., Zheng J., Wang C., Chan W.C.W. (2006). Optimizing the synthesis of red- to near-ir-emitting cds-capped cdtexse1-x alloyed quantum dots for biomedical imaging. Chem. Mater.

[133] Wang X.Y., Qu L.H., Zhang J.Y., Peng X.G., Xiao M. (2003). Surface-related emission in highly luminescent cdse quantum dots. Nano Lett.

[134] Talapin D.V., Mekis I., Gotzinger S., Kornowski A., Benson O., Weller H. (2004). Cdse/Cds/Zns and Cdse/Znse/Zns core-shell-shell nanocrystals. J. Phys. Chem. B.

[135] Chan W.C.W., Nie S.M. (1998). Quantum dot bioconjugates for ultrasensitive nonisotopic detection. Science.

[136] Gopalakrishnan G., Danelon C., Izewska P., Prummer M., Bolinger P.Y., Geissbuehler I., Demurtas D., Dubochet J., Vogel H. (2006). Multifunctional lipid/quantum dot hybrid nanocontainers for controlled targeting of live cells. Angew. Chem.—Int. Edition.

[137] Kairdolf B.A., Mancini M.C., Smith A.M., Nie S. (2008). Minimizing nonspecific cellular binding of quantum dots with hydroxyl-derivatizied surface coatings. Anal. Chem..

[138] Lau P.C., Norwood R.A., Mansuripur M., Peyghambarian N. (2013). An effective and simple oxygen nanosensor made from mpa-capped water soluble cdte nanocrystals. Nanotechnology.

[139] Zhang H., Cui Z.C., Wang Y., Zhang K., Ji X.L., Lu C.L., Yang B., Gao M.Y. (2003). From water-soluble cdte nanocrystals to fluorescent nanocrystal-polymer transparent composites using polymerizable surfactants. Adv. Mater.

[140] Li L., Qian H.F., Ren J.C. (2005). Rapid synthesis of highly luminescent Cdte nanocrystals in the aqueous phase by microwave irradiation with controllable temperature. Chemi. Commun..

[141] He Y., Lu H.T., Sai L.M., Lai W.Y., Fan Q.L., Wang L.H., Huang W. (2006). Synthesis of Cdte nanocrystals through program process of microwave irradiation. J. Phys. Chem. B.

[142] He Y., Sai L.M., Lu H.T., Hu M., Lai W.Y., Fan Q.L., Wang L.H., Huang W. (2007). Microwave-assisted synthesis of water-dispersed cdte nanocrystals with high luminescent efficiency and narrow size distribution. Chem. Mater.

[143] He Y., Lu H.T., Sai L.M., Su Y.Y., Hu M., Fan C.H., Huang W., Wang L.H. (2008). Microwave synthesis of water-dispersed Cdte/Cds/Zns core-shell-shell quantum dots with excellent photostability and biocompatibility. Adv. Mater.

[144] Yuwen L., Bao B., Liu G., Tian J., Lu H., Luo Z., Zhu X., Boey F., Zhang H., Wang L. (2011). One-pot encapsulation of luminescent quantum dots synthesized in aqueous solution by amphiphilic polymers. Small.

[145] Yuwen L., Lu H., He Y., Chen L., Hu M., Bao B., Boey F., Zhang H., Wang L. (2010). A facile low temperature growth of cdte nanocrystals using novel dithiocarbamate ligands in aqueous solution. J. Mater. Chem..

[146] Xing Y., Chaudry Q., Shen C., Kong K.Y., Zhau H.E., Wchung L., Petros J.A., O’Regan R.M., Yezhelyev M.V., Simons J.W. (2007). Bioconjugated quantum dots for multiplexed and quantitative immunohistochemistry. Nat. Protoc.

[147] Hu M., He Y., Song S., Yan J., Lu H.T., Weng L.X., Wang L.H., Fan C. (2010). DNA-bridged bioconjugation of fluorescent quantum dots for highly sensitive microfluidic protein chips. Chem. Commun.

[148] Hu M., Yan J., He Y., Lu H., Weng L., Song S., Fan C., Wang L. (2010). Ultrasensitive, multiplexed detection of cancer biomarkers directly in serum by using a quantum dot-based microfluidic protein chip. ACS Nano.

[149] Kim J., Seo K., Wang J. (2004). Multiplexed electrochemical protein coding based on quantum dot (qd)-bioconjugates for a clinical barcode system. Conf. Proc. IEEE Eng. Med. Biol. Soc.

[150] Ma H.L., Wang L.P., Li W., Xu S.K. (2004). Fret study of Qd protein bioconjugates. J. Pept. Sci.

[151] Vinayaka A.C., Thakur M.S. (2011). Photoabsorption and resonance energy transfer phenomenon in cdte-protein bioconjugates: An insight into qd-biomolecular interactions. Bioconj. Chem..

[152] Bakalova R., Zhelev Z., Ohba H., Baba Y. (2005). Quantum Dot-based Western Blot technology for ultrasensitive detection of tracer proteins. J. Am. Chem. Soc.

[153] Shingyoji M., Gerion D., Pinkel D., Gray J.W., Chen F.Q. (2005). Quantum Dots-based reverse phase protein microarray. Talanta.

[154] Ligler F.S., Sapsford K.E., Golden J.P., Shriver-Lake L.C., Taitt C.R., Dyer M.A., Barone S., Myatt C.J. (2007). The array biosensor: Portable, automated systems. Anal. Sci..

[155] Yan J., Hu M., Li D., He Y., Zhao R., Jiang X., Song S., Wang L., Fan C. (2008). A nano- and micro- integrated protein chip based on quantum dot probes and a microfluidic network. Nano Res.

[156] Sapsford K.E., Berti L., Medintz I.L. (2006). Materials for fluorescence resonance energy transfer analysis: beyond traditional donor-acceptor combinations. Angew. Chem.—Int. Edition.

[157] Jares-Erijman E.A., Jovin T.M. (2003). Fret imaging. Nat. Biotechnol.

[158] Miyawaki A., Sawano A., Kogure T. (2003). Lighting up cells: Labelling proteins with fluorophores. Nat. Cell Biol..

[159] Akinfieva O., Nabiev I., Sukhanova A. (2013). New directions in quantum dot-based cytometry detection of cancer serum markers and tumor cells. Crit. Rev. Oncol. Hematol.

[160] Bao B., Yuwen L., Zheng X., Weng L., Zhu X., Zhan X., Wang L. (2010). A fluorescent conjugated polymer for trace detection of diamines and biogenic polyamines. J. Mater. Chem..

[161] Li M., Zhou X., Guo S., Wu N. (2013). Detection of lead (ii) with a “turn-on” fluorescent biosensor based on energy transfer from Cdse/Zns quantum dots to graphene oxide. Biosens. Bioelectron.

[162] He Y., Xiong L.H., Xing X.J., Tang H.W., Pang D.W. (2013). An ultra-high sensitive platform for fluorescence detection of micrococcal nuclease based on graphene oxide. Biosens. Bioelectron.

[163] Dong H., Gao W., Yan F., Ji H., Ju H. (2010). Fluorescence resonance energy transfer between quantum dots and graphene oxide for sensing biomolecules. Anal. Chem.

[164] Loh K.P., Bao Q., Eda G., Chhowalla M. (2010). Graphene oxide as a chemically tunable platform for optical applications. Nat. Chem.

[165] Hu K., Liu J., Chen J., Huang Y., Zhao S., Tian J., Zhang G. (2013). An amplified graphene oxide-based fluorescence aptasensor based on target-triggered aptamer hairpin switch and strand-displacement polymerization recycling for bioassays. Biosens. Bioelectron.

[166] Liang J., Wei R., He S., Liu Y., Guo L., Li L. (2013). A highly sensitive and selective aptasensor based on graphene oxide fluorescence resonance energy transfer for the rapid determination of oncoprotein Pdgf-Bb. Analyst.

[167] Lin M.-Y., Lu Y.-P., Grumezescu A.M., Ho F.H., Kao Y.H., Yang Y.S., Yang C.H. (2013). Tumor marker detection by aptamer-functionalized graphene oxide. Curr. Org. Chem..

[168] Liu Q., Xu X., Zhang L., Luo X., Liang Y. (2013). Assembly of single-stranded polydeoxyadenylic acid and beta-glucan probed by the sensing platform of graphene oxide based on the fluorescence resonance energy transfer and fluorescence anisotropy. Analyst.

[169] Yu Y., Cao Q., Zhou M., Cui H. (2013). A novel homogeneous label-free aptasensor for 2,4, 6-trinitrotoluene detection based on an assembly strategy of electrochemiluminescent graphene oxide with gold nanoparticles and aptamer. Biosens. Bioelectron.

[170] Zhu C., Zeng Z., Li H., Li F., Fan C., Zhang H. (2013). Single-layer mos2-based nanoprobes for homogeneous detection of biomolecules. J. Am. Chem. Soc.

[171] Tu Y., Li W., Wu P., Zhang H., Cai C. (2013). Fluorescence quenching of graphene oxide integrating with the site-specific cleavage of the endonuclease for sensitive and selective microrna detection. Anal. Chem.

[172] Pei H., Li J., Lv M., Wang J., Gao J., Lu J., Li Y., Huang Q., Hu J., Fan C. (2012). A graphene-based sensor array for high-precision and adaptive target identification with ensemble aptamers. J. Am. Chem. Soc.

[173] Geissler D., Stufler S., Loehmannscroeben H.G., Hildebrandt N. (2013). Six-color time-resolved forster resonance energy transfer for ultrasensitive multiplexed biosensing. J. Am. Chem. Soc.

[174] Watanabe M., Kajikawa K. (2003). An optical fiber biosensor based on anomalous reflection of gold. Sens. Actuators B.

[175] Chang Y.F., Hung S.H., Lee Y.J., Chen R.C., Su L.C., Lai C.S., Chou C. (2011). Discrimination of breast cancer by measuring prostate-specific antigen levels in women’s serum. Anal. Chem.

[176] Healy D.A., Hayes C.J., Leonard P., McKenna L., O’Kennedy R. (2007). Biosensor developments: Application to prostate-specific antigen detection. Trends Biotechnol.

